# Metabolomic Profiling Uncovers Physiological and Parasitological Adaptations to Bee Pollen Supplementation in Goats

**DOI:** 10.3390/biology15191717

**Published:** 2026-09-29

**Authors:** Adriana Rojas, Lauren K. Solice, Reagan N. Sims, Brian L. Bruner, Adeyemi A. Olanrewaju, Rodolfo C. Cardoso, Morteza Hosseini Ghaffari, Cesar A. Rosales-Nieto

**Affiliations:** 1 Department of Agricultural Sciences, Texas State University, San Marcos, TX 78666, USA; 2 Medical Laboratory Science Program, Texas State University, San Marcos, TX 78666, USA; 3 Department of Animal Science, Texas A&M University, College Station, TX 77843, USA; 4 Research Institute for Farm Animal Biology (FBN), 18196 Dummerstorf, Germany

**Keywords:** bee products, biochemical markers, hepatic function, anemia, fparasitic infections

## Abstract

Parasitic infections compromise livestock health, productivity, and profitability, while rising anthelmintic resistance restricts the effectiveness of conventional parasite control strategies. Bee pollen (BP) contains bioactive compounds with immunomodulatory and antimicrobial potential. This study investigated whether dietary BP supplementation reduces parasite burden and influences liver function, hematological and metabolic profiles, and circulating metabolomic signatures in goats. Thirty crossbred Boer × Spanish does were randomly assigned to either a BP-supplemented group (T-BP; n = 15) or a control group (T-CTL; n = 15). Over 49 days, T-BP goats received 10 g/day BP mixed with 50 g/day pelleted feed, while controls received the same feed without BP. Body weight, weekly blood samples, FAMACHA scores, and biweekly fecal egg counts (FEC) were collected. We assessed metabolomic profiles on days −8, 21, 42, and 56. Data were analyzed using mixed models and repeated measures. We identified strongyle-type eggs, presumably *Haemonchus contortus*, as the predominant parasite. No significant differences were observed between treatments for body weight, liver enzymes, metabolic biomarkers, or hematological profiles (*p* > 0.05). FEC varied over time (*p* < 0.001), with significantly lower FEC in BP-supplemented goats only on Day 42 (*p* < 0.05). Metabolomic differences were significant on Days 21 and 42 but not on Day 56. These differences involved metabolites annotated to lipid signaling, inflammatory processes, and cellular energy metabolism. In summary, BP supplementation demonstrated a potential antiparasitic effect against *H. contortus* but did not significantly affect liver, metabolic, or hematological parameters. Metabolomic findings should be considered exploratory and hypothesis-generating.

## Simple Summary

Bee pollen (BP) contains bioactive compounds that may influence host physiology, but evidence in adult ruminants remains limited. This study evaluated 10 g/day BP in crossbred Boer × Spanish does during a 42-day supplementation period following a 7-day adaptation. Body weight, hematological and hepatic biomarkers, FAMACHA scores, fecal egg counts (FEC), and plasma metabolomic profiles were evaluated. We identified strongyle-type eggs, presumably *Haemonchus contortus*, as the predominant parasite. BP did not produce consistent treatment effects on body weight, hepatic biomarkers, hematological indices, FAMACHA scores, or FEC across the study period. FEC fluctuated over time; BP supplementation did not consistently reduce these counts, although the BP group showed lower values at the end of the study. Metabolomic differences were detected during supplementation (Days 21 and 42) but were not evident 14 days after supplementation ended (Day 56). These metabolomic findings are exploratory and hypothesis-generating; larger dose-ranging studies with prespecified longitudinal analyses are needed.

## 1. Introduction

Nutritional strategies that improve metabolic efficiency, health, and disease resilience are increasingly important in sustainable and precision livestock systems. Natural bioactive compounds have gained attention as alternatives to synthetic additives, driven by concerns about antimicrobial resistance, environmental impact, and consumer demand for “clean-label” products. Bee pollen (BP) is one such candidate, notable for its dense nutritional composition and diverse pharmacological properties, including antioxidant, anti-inflammatory, hepatoprotective, and immunomodulatory activities [1,2,3,4]. Although BP’s chemical profile varies by geographic and botanical origin [5], it generally contains proteins, essential amino acids, polyunsaturated fatty acids, vitamins, minerals, polyphenols, and flavonoids [4]. These constituents have been reported to improve antioxidant status, modulate hepatic enzyme activity, enhance erythropoiesis, and improve nutrient digestibility in monogastrics (poultry, rodents, rabbits) and pre-ruminant calves but not adult ruminants [6,7,8,9,10].

Despite the growing body of evidence, the biological actions of BP in ruminants remain poorly understood. Ruminants have a fermentative digestive system that may facilitate the degradation of BP’s recalcitrant outer wall [11], potentially enhancing bioavailability compared with monogastrics [12]. Therefore, results from monogastric models or pre-ruminant calves cannot be directly extrapolated to adult goats. In ruminants, nutritional interventions that support hepatic and metabolic homeostasis can enhance productivity, reproductive efficiency, and resilience to physiological and environmental stressors [13]. However, systematic evaluations of BP supplementation in goats are scarce, and existing studies on ewes report inconsistent outcomes regarding antioxidant capacity, hematological parameters, and productive parameters [14]. Dose–response relationships and mechanistic pathways remain largely undefined.

This knowledge gap is especially consequential for goats, a species of major global importance for their adaptability and multifunctionality in meat, milk, and fiber production [15]. In many semi-arid and tropical regions, goat performance is constrained by nutritional stress and endemic parasitism, particularly infections with *Haemonchus contortus* (*H. contortus*) and *Eimeria* spp., pathogens responsible for anemia, impaired growth, gastrointestinal dysfunction, and substantial economic losses [16,17,18]. Recent surveys during Fall and Winter in the United States report high burdens of *H. contortus* and *Trichostrongylus*, even in dry, forage-limited regions [19], while *Eimeria* spp. remain prevalent across North America [20]. With anthelmintic resistance now widespread [21], developing nutritional strategies that enhance immune competence, hematological resilience, and tolerance to parasitic challenge is increasingly urgent. Evidence from monogastrics suggests that BP may modulate leukocyte profiles, stimulate erythropoiesis, and bolster antioxidant defenses [4,10], yet comparable data in goats are lacking.

Central to evaluating the physiological and health-related impacts of BP are hematological profiles, parasitological indicators, and serum metabolic biomarkers. Complete blood count (CBC) traits are commonly used as indicators of immune competence, inflammatory responses, and overall physiological condition by assessing animals’ capacity for oxygen transport, immune defense, and blood clotting. Aligned with CBC, the FAMACHA score reflects anemia severity associated with blood-feeding gastrointestinal parasites. Fecal egg counts (FECs) are commonly used as an indirect indicator of gastrointestinal parasite burden. Circulating biomarkers such as alanine aminotransferase (ALT), aspartate aminotransferase (AST), alkaline phosphatase (ALP), gamma-glutamyl transferase (GGT), albumin, total protein, and bilirubin provide sensitive indices of hepatic integrity, protein metabolism, and systemic energy balance [22]. Together, these indicators offer a multidimensional framework for assessing whether BP modulates physiological processes relevant to resilience under field conditions. Additionally, a metabolomic profile provides clear information about an organism’s status and can help clarify the molecular mechanisms and pathways associated with BP supplementation and the animal model’s pathophysiological state [23]. We hypothesized that BP’s diverse bioactive constituents would improve goat health through the regulation of lipid metabolism and immune-related pathways and that metabolomic profiles could provide correlative evidence.

## 2. Materials and Methods

The Institutional Animal Care and Use Committee of Texas State University approved the animal procedures in this study (IACUC #9157). The procedures comply with the ARRIVE guidelines for reporting animal research [24].

The study was conducted at the Goat Unit of Texas State University, located at Freeman Ranch (29°56′10.79″ N, 98°00′18.00″ W), where the climate features hot summers and mild winters. Precipitation varies throughout the year, with May typically receiving the most rainfall. During the study period (May–July), temperatures generally ranged from 30 °C to 34 °C. Temperatures, precipitation, and humidity were above average during the study period. Rainfall during this period totalled 181.6 mm, with maximum and minimum temperatures of 38.8 °C and 10.0 °C, respectively. The recorded humidity was approximately 70%.

### 2.1. Experimental Design

**Figure 1:**
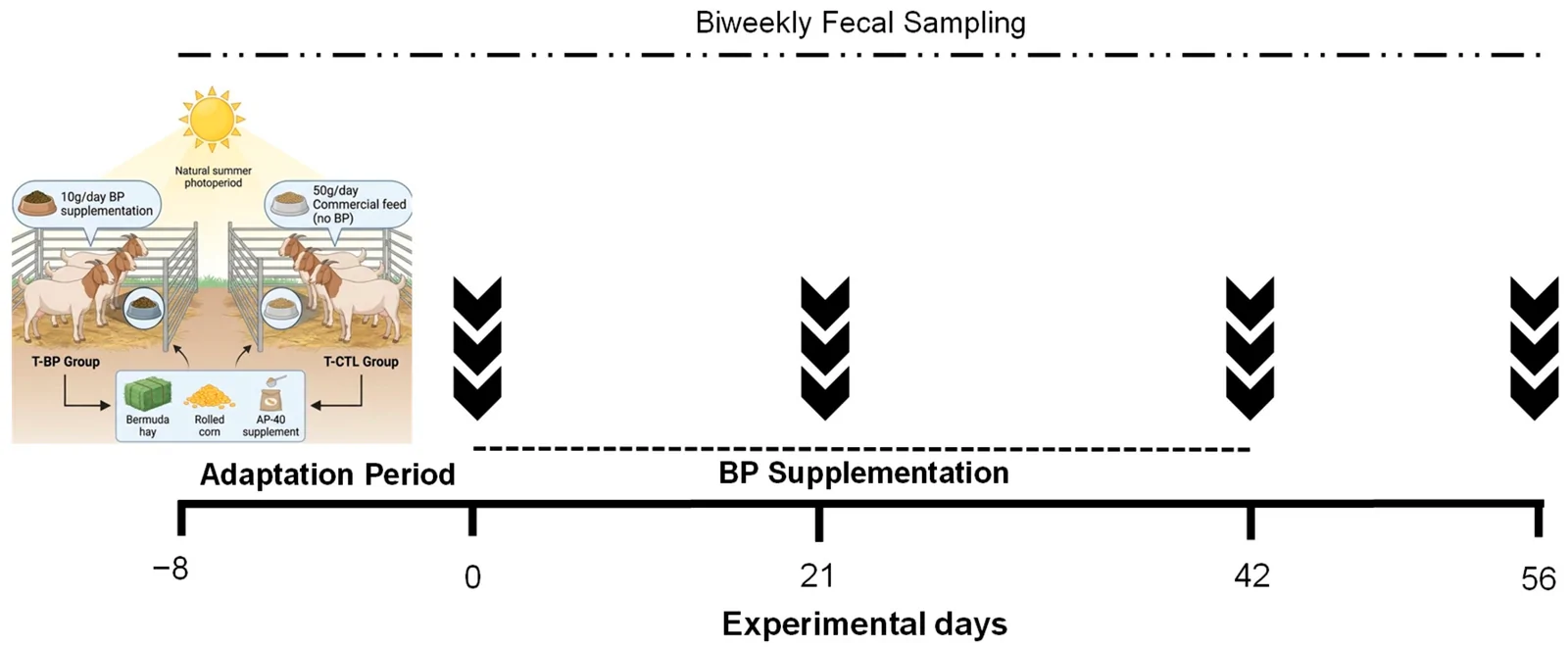
Schematic of experimental design of the study cohort during summer. Following the 7-day adaptation period, T-BP received 10 g of BP for 42 days, whereas T-CTL received the carrier feed without BP (Dashed line). Both groups had access to a maintenance ration of Bermuda hay, rolled corn and pellets (AP−40; Angelo Inc^®^). Solid lines represent weekly live weight, blood samples, and FAMACHA assessments. Dash dot line represents biweekly fecal assessment. Arrows represent plasma metabolomic samples collected at Day-8, 21, 42, and 56.

To assess the effects of BP supplementation on hepatic function, metabolic biomarkers, hematological parameters [CBC]), gastrointestinal load (FECs), and metabolomic signature, we used 30 crossbred Spanish × Boer females. Animals were assigned to one of two groups (n = 15 per group) based on initial live weight: T-BP received 10 g BP mixed with 50 g of commercial feed, and T-CTL received 50 g of the same feed without BP. All goats underwent a 7-day adaptation period followed by 42 days of supplementation (Figure 1). To ensure complete intake, goats were individually fed in the milking parlor. BP was obtained from a commercial source (Walker Honey Farm, Rogers, TX, USA), and its chemical composition was analyzed by SDK Laboratories (Hutchinson, KS, USA; Supplementary Table S1). Because research on BP use in goats is limited and no dose-titration study was available, we used the trial dose from a previous ruminant study [14]. All goats received a basal maintenance diet formulated to meet NRC requirements (see Section 2.2).

### 2.2. Nutritional Diet

A total mixed ration was offered in feeders providing 30 cm of linear space per goat to minimize competition and ensure unrestricted access to feed. The diets were formulated to meet the nutritional requirements of adult, non-pregnant goats with low physical activity, so that any differences observed could be attributed to BP supplementation rather than diet or exercise [25]. The diet was designed to reflect the semi-arid conditions of South Texas and comparable regions, characterized by variable rainfall and sparse vegetation [26]. Both groups received the same basal maintenance ration of Bermuda hay, rolled corn, and a commercial supplement (AP-40, Angelo Inc^®^, San Angelo, TX, USA). T-BP additionally received 10 g/day BP, whereas T-CTL received carrier feed alone; because the basal ration was not adjusted to compensate for BP nutrients, the complete treatment diets were not strictly isoproteic or isoenergetic. The AP-40 specification was reported as 9% crude protein and 32 MJ metabolizable energy, but the energy basis (e.g., per kg dry matter) requires verification from the product specification. All feed was consumed, and no residual feed was collected. We included only clinically healthy animals, and all goats had continuous access to clean water and mineral and salt blocks.

### 2.3. Measurements

#### 2.3.1. Body Weight

To assess the effect of BP on daily body weight gain, we recorded body weights weekly during the trial (Figure 1) using a mobile scale with a 1000 kg capacity and a precision of 1 kg (W-W Paul Scales, model 58SX; Duncan, OK, USA). Daily weight gain (DWG) was calculated based on the initial and final weights divided by the number of experimental days.

#### 2.3.2. Hepatic Biomarkers

To evaluate hepatic biomarkers, we collected blood samples weekly from all goats in both treatment groups via jugular venipuncture using lithium heparin tubes (BD Vacutainer^®^, Preanalytical Solutions, Franklin Lakes, NJ, USA). We acclimated animals to handling procedures eight days before the start of the trial to reduce stress-induced variability in physiological measurements. Immediately after collection, we placed blood samples on ice and transported them to the laboratory, where we centrifuged them at 2000 rpm for 15 min. We separated plasma and stored it at −20 °C until analysis.

Key plasma biomarkers included total protein, albumin, and bilirubin, which reflect protein metabolism, hepatic function, and potential hepatotoxicity. Liver integrity was also assessed by measuring plasma concentrations of hepatic enzymes: alanine aminotransferase (ALT), aspartate aminotransferase (AST), alkaline phosphatase (ALP), and gamma-glutamyl transferase (GGT). All biomarkers were quantified using commercially available ELISA or colorimetric assay kits (MyBioSource, San Diego, CA, USA), following the manufacturers’ protocols to ensure analytical sensitivity and specificity. We chose sampling time points to capture baseline values and potential acute or delayed treatment effects. Not all biomarkers were analyzed at every time point due to budget constraints. Total protein (TP) and liver enzymes (ALT, AST, ALP, GGT) were assessed on three dates (day-8 [baseline], 21 [mid], and 42 [end]), albumin on four occasions (day-8, 14, 28, and 42), and bilirubin on all dates.

Total Protein (TP; Catalog #MBS2540455) was assessed using a bicinchoninic acid (BCA)-based assay (λ = 562 nm). TP concentrations were calculated from the standard curve using the formula: (OD_sample − OD_blank)/(OD_standard − OD_blank) × 563 µg/mL × dilution factor. The assay demonstrated a linear range of 20–200 µg/mL (R^2^ = 0.999), with intra- and inter-assay CVs of 2.06% and 4.72%, respectively. ALT (Catalog #MBS8819949) concentration was assessed at 450 nm with a detection range of 1.57–100 U/L and sensitivity of 0.63 U/L. AST (Catalog #MBS8819952) concentration was assessed at 450 nm, detection range 0.16–10 U/L, sensitivity 0.055 U/L. ALP (Catalog #MBS2800395) concentration was assessed at 540 nm, with a detection range of 1.56–100 ng/mL. The standard curve included eight concentration points (0–100 ng/mL). Intra- and inter-assay CVs were <8% and <12%, respectively. GGT (Catalog #MBS1603612) concentration was assessed at 450 nm with a detection range of 0.3–90 ng/mL and sensitivity of 0.13 ng/mL. Intra- and inter-assay CVs were <8% and <10%, respectively. Standard curve fitting and interpolation were conducted using Curve Expert 1.4 software for all ELISA-based enzyme assays. Albumin (Catalog #MBS8305386) concentration was assessed using a bromocresol purple (BCP) dye-binding colorimetric assay (λ = 610 nm). The standard curve followed the equation *y* = 0.0817*x*^0.4013 (R^2^ = 0.9958). Bilirubin (Catalog #MBS169250) concentration was assessed at 540 nm with a detection sensitivity of 0.5 mg/dL.

#### 2.3.3. Hematological Analysis—Complete Blood Count (CBC)

To evaluate hematological health and potential immunological effects of BP supplementation, clinical scoring and laboratory techniques were used throughout the study. Goats from both treatment groups were assessed weekly using CBC. Whole blood samples were collected weekly via jugular venipuncture using 21-gauge, 1½-inch needles into EDTA-coated tubes (BD Vacutainer^®^, Preanalytical Solutions, Franklin Lakes, NJ, USA). Samples were immediately placed on ice and transported to the laboratory for CBC analysis. CBCs were conducted using a veterinary hematology analyzer (Vetscan HM5, Zoetis Diagnostics, Parsippany, NJ, USA) to evaluate hematological profiles. Parameters measured included white blood cells (WBC), lymphocytes (LYM), monocytes (MON), neutrophils (NEU), red blood cells (RBC), hemoglobin (HGB), mean corpuscular volume (MCV), mean corpuscular hemoglobin (MCH), and mean corpuscular hemoglobin concentration (MCHC). These values were used to assess whether BP supplementation influenced immune response, anemia, inflammation, or resilience to parasites.

#### 2.3.4. FAMACHA Scoring, and Fecal Egg Counts (FECs)

FAMACHA scoring was conducted weekly by trained personnel to estimate the level of anemia based on the color of the lower eyelid mucous membrane [27]. Scores ranged from 1 (optimal) to 5 (severely anemic) and were used as a visual indicator of the animals’ anemia status, particularly in relation to *H. contortus* infections [27]. We performed FAMACHA evaluations weekly to monitor both parasite-related symptoms and the potential effects of BP supplementation.

Fecal samples (FECs) were collected directly from the rectum of each goat and processed within one hour. We used the modified McMaster technique [28,29] to quantify FECs. We prepared a flotation solution by mixing sodium chloride and water at a 1:7 ratio, resulting in a specific gravity of 1.23 g/mL. Two grams of feces were homogenized in 12 mL of the solution, or the volume was adjusted when a different fecal mass was used (×14 dilution factor). After a 5-min settling period and filtration through a strainer to remove coarse material, the sample was left undisturbed for another 5 min. A disposable pipette was used to draw the upper fluid layer, which was dispensed into three chambers of McMaster slides. Slides were examined under 10× magnification, and eggs within each chamber grid were counted. Egg per gram (EPG) of feces were calculated using the following formula: EPG = number of eggs counted × dilution factor × volume of flotation solution/fecal weight (g). This method allowed quantitative tracking as an indirect indicator of gastrointestinal nematode infection intensity.

#### 2.3.5. Metabolomic Profiles

Metabolomic sampling was performed at four prespecified time points: baseline before treatment; Day-8), mid-experiment (Day 21), end-experiment (Day 42), and after supplementation ended (Day 56) in 10 animals per treatment group were randomly from each experimental group comparisons were interpreted descriptively and were considered consistent with a treatment group.

Samples were thawed on ice, and 100 μL of each sample was transferred into a tube, then 300 μL of methanol was added. Vortexed all samples for 30 s, then sonicated for 30 min at 4 °C. Each sample was kept at −20 °C for 1 h, then vortexed for 30 s and kept at 4 °C for 15 min. Samples were then centrifuged at 12,000 rpm and 4 °C for 10 min. Finally, 200 μL of supernatant and 5 μL of DL-o-Chlorophenylalanine (0.14 mg/mL) were transferred to a vial for LC-MS analysis. Quality-control (QC) samples were prepared by pooling equal aliquots of extract from all study samples and were processed using the same sample-preparation procedure as the study samples. The pooled QC was used to monitor overall analytical performance during LC-MS acquisition. Because additional quantitative QC acceptance metrics (e.g., feature-level QC RSD thresholds) were not available in the analytical record, no such thresholds are reported or inferred.

#### 2.3.6. Statistical Analysis

We performed statistical analyses using SAS version 9.4 [30]. We assessed normality with Proc Univariate. We treated each goat as the experimental unit. Plasma albumin, bilirubin, TP, hepatic enzymes (ALT, AST, ALP, GGT), CBC parameters, FEC, and FAMACHA scores were analyzed using the MIXED procedure by date and for repeated observations within goat. Body weight measured at the time of sampling as a covariate in the main effects of treatment and time, as well as their interaction. We estimated correlations among continuous variables using PROC GLM with the MANOVA option. We set statistical significance at *p* ≤ 0.05, with 0.05 < *p* ≤ 0.10. Data are presented as mean ± SEM.

For metabolomic profiling, raw data were acquired and aligned in Compound Discoverer (3.0, Thermo) using *m*/*z* and retention time information. Features from ESI− or ESI+ were merged and imported into SIMCA-P (version 14.1). PCA was used for unsupervised visualization and outlier assessment, and between-group differences in multivariate space were evaluated using PERMANOVA with 999 permutations. PLS-DA/OPLS-DA) was used to identify potential biomarkers. Differential metabolites were defined using |log2FC| > 1 and FDR-adjusted *p* < 0.05; inference therefore emphasized PCA, PERMANOVA, and FDR-controlled metabolite-level comparisons.

## 3. Results

### 3.1. Effect of BP on Body Weight Gains

**Figure 2:**
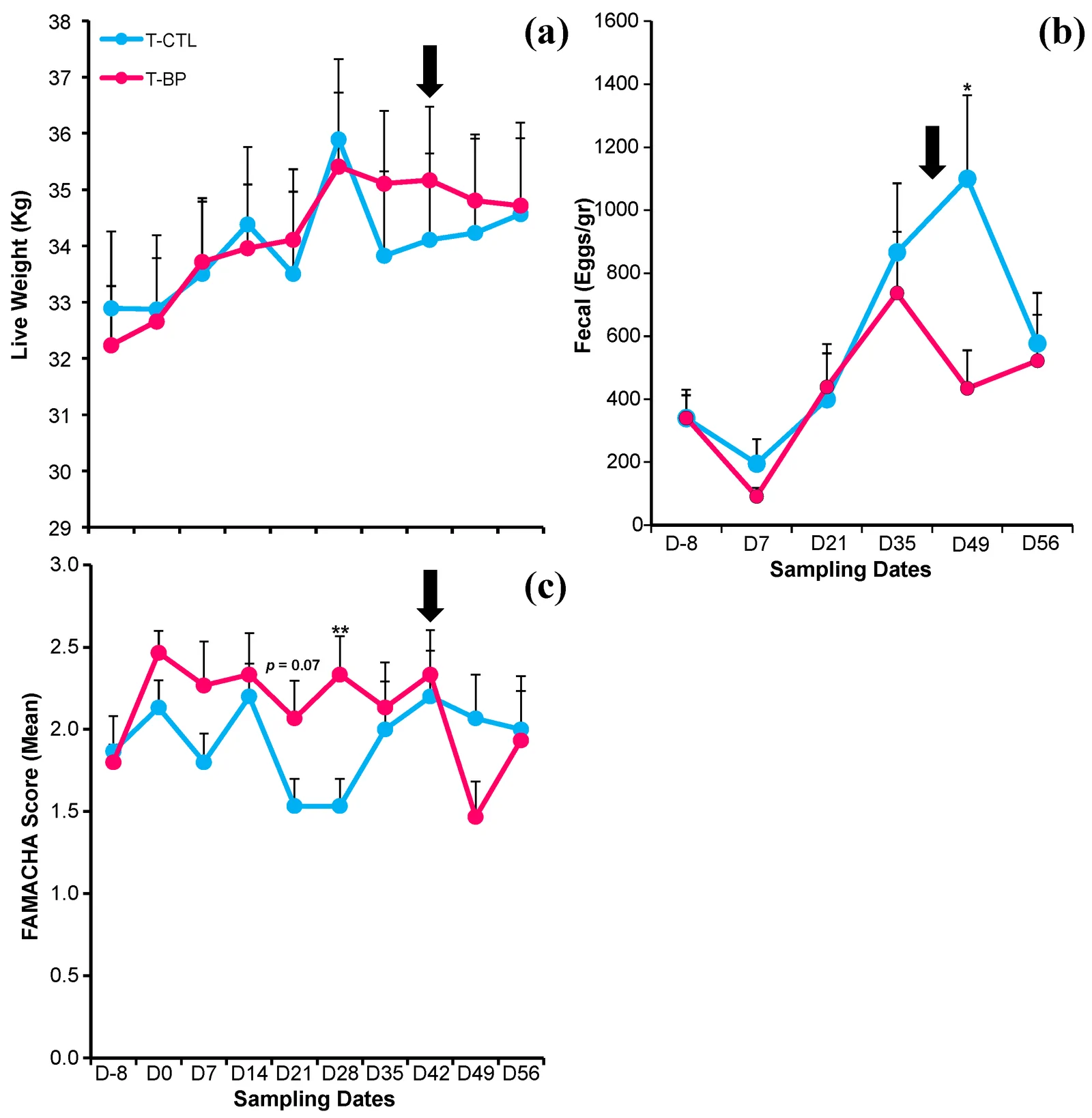
(**a**) Body weight, (**b**) fecal counts, and (**c**) FAMACHA Scores in crossbreed Spanish*Boer goats supplemented with BP at a dose of 10 g/day/head (T-BP; pink line) or without supplementation (T-CTL; blue line) for 42 d during the suboptimal breeding season. Sampling date D-8 represents the basal date. Sampling date D21 represents the middle of the experiment, and sampling date D42 represents the end of the supplementation (Dark arrow). Values are presented in mean ± SEM. * *p* < 0.05; ** *p* < 0.01.

Initial and final body weight and average daily gain did not differ between treatments (*p* > 0.05; Figure 2a). Body weight differed across time (*p* < 0.01; Figure 2a).

### 3.2. Effect of BP on Parasitological Parameters

FAMACHA scores fluctuated in both treatments but were similar between them (*p* > 0.05). FAMACHA scores differed across time (*p* < 0.01; Figure 2b). FAMACHA scores were higher in goats from the T-BP in the sample on D21 (*p* = 0.07), and D28 (*p* < 0.01), but not at the other sampling dates (*p* > 0.05; Figure 2b). Interactions between treatment and sampling date, were not significant (*p* > 0.05).

FECs increased in both groups toward the end of the experiment but were similar between treatments (*p* > 0.05). FECs differed across time (*p* < 0.01; Figure 2c). We identified strongyle-type eggs, presumably *Haemonchus contortus*, as the predominant parasite of all samples. Low-level infections with *Trichuris* spp. and *Eimeria* spp. were occasionally detected, but their prevalence and intensity were minimal and insufficient for inclusion in quantitative analysis. FEC was higher in the T-CTL group at the isolated late sampling point reported in Figure 2c (*p* < 0.05), but not at the other sampling dates (*p* > 0.05). The treatment × sampling date, were not significant (*p* > 0.05).

### 3.3. Effect of BP on Hematological Parameters

#### 3.3.1. White Blood Cells

**Figure 3:**
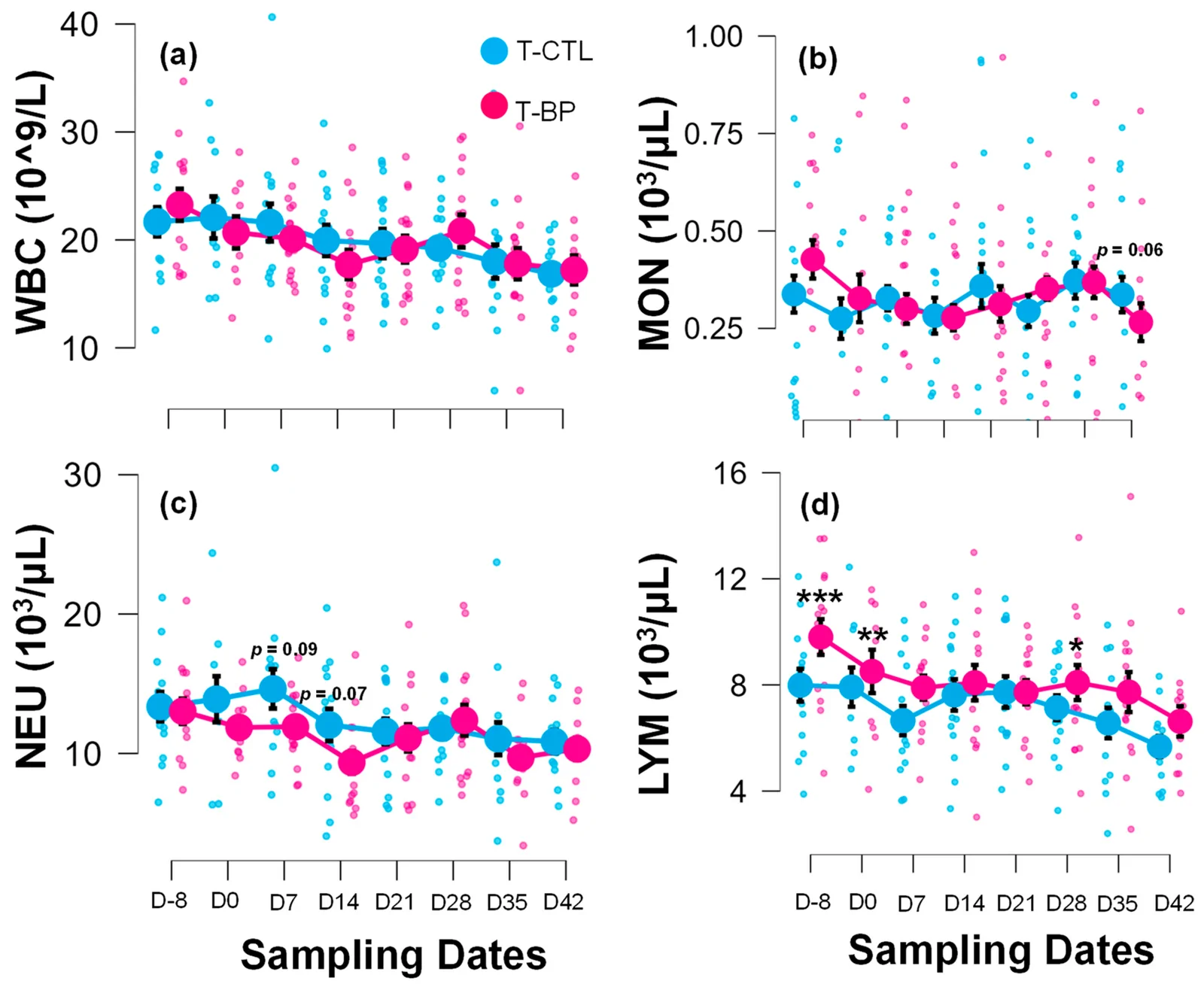
White blood cell biomarkers from goats supplemented with Bee Pollen or not. (**a**) Circulating concentrations of white blood cells (WBC), (**b**) Monocyte (MON), (**c**) Neutrophil (NEU), and (**d**) Lymphocytes (LYM) in crossbreed Spanish*Boer goats supplemented with BP at a dose of 10 g/day/head (T-BP; pink line) or without supplementation (T-CTL; blue line) for 42 d during the suboptimal breeding season. Values are presented in mean ± SEM. * *p* < 0.05; ** *p* < 0.01; *** *p* < 0.001.

WBC concentrations decreased in both treatments and differed between groups (*p* < 0.001), with significant variation over time (*p* < 0.001; Figure 3a). Body weight positively influenced WBC concentrations, increasing by 0.7^10^9/L^ per 10 kg increase in body weight (*p* < 0.05). The treatment × sampling date interaction was not significant (*p* > 0.05).

NEU concentrations decreased in both groups (*p* < 0.001), differed between treatments (*p* < 0.01), and varied over time (*p* < 0.001; Figure 3b). NEU concentrations tended to be higher in T-CTL goats on D7 (*p* = 0.09) and D14 (*p* = 0.07), with no differences at other dates. The treatment × sampling date interaction was not significant (*p* > 0.05).

MON concentrations remained stable over time within treatments (*p* = 0.07) and did not differ between treatments (*p* > 0.05), although they tended to vary across sampling dates (*p* = 0.06; Figure 3c). MON concentrations tended to be higher in T-CTL goats on D42 (*p* = 0.06), with no differences at other dates. The treatment × sampling date interaction was not significant (*p* > 0.05).

LYM concentrations decreased in T-BP goats but increased in T-CTL goats (*p* < 0.01) and varied over time (*p* < 0.001; Figure 3d). LYM concentrations were higher in T-BP goats on D−8 (*p* < 0.001), D0 (*p* < 0.01), and D28 (*p* < 0.05), with no differences at other dates. The treatment × sampling date interaction was not significant (*p* > 0.05).

#### 3.3.2. Red Blood Cell Count Values

**Figure 4:**
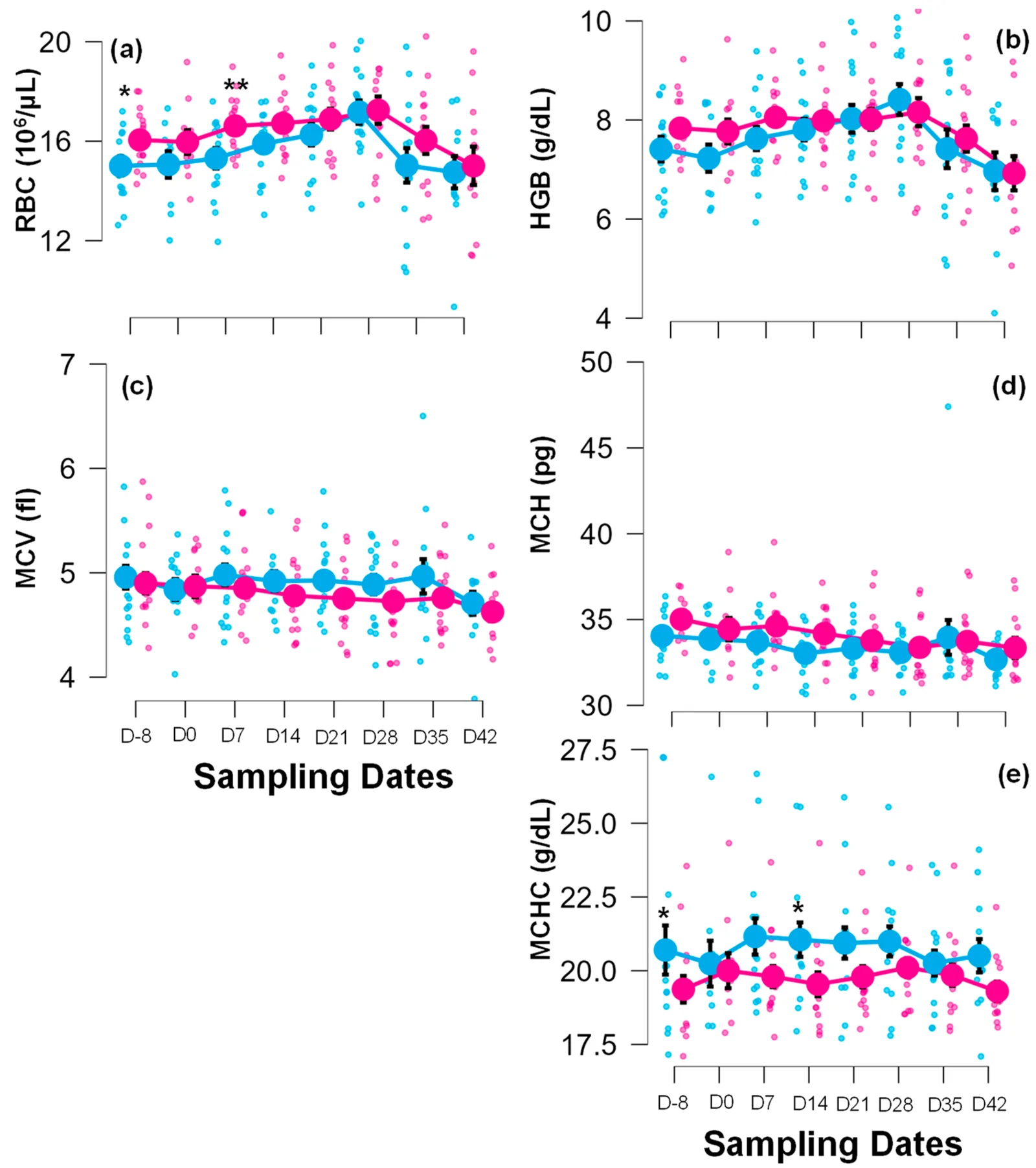
Anemia response biomarkers from goats supplemented with Bee Pollen or not. Mean (±) circulating concentrations of red blood cells (RBC; (**a**)), hemoglobin (HGB; (**b**)) mean corpuscular volume (MCV; (**c**)), mean corpuscular hemoglobin (MCH; (**d**)), and mean corpuscular hemoglobin (MCH; (**e**)) in crossbreed Spanish*Boer goats supplemented with BP at a dose of 10 g/day/head (T-BP; pink line) or without supplementation (T-CTL; blue line) for 42 d during the suboptimal breeding season. * *p* < 0.05; ** *p* < 0.01.

RBC concentrations fluctuated over time (*p* < 0.001) but did not differ between treatments overall (*p* > 0.05; Figure 4a). RBC concentrations were higher in T-BP goats on D-8 (*p* < 0.05) and D7 (*p* < 0.01), with no differences at other dates. The treatment × sampling date interaction tended toward significance (*p* = 0.06).

HGB concentrations differed between treatments (*p* < 0.05) and over time (*p* < 0.001; Figure 4b). The treatment × sampling date interaction approached significance (*p* = 0.06).

MCV concentrations differed between treatments (*p* < 0.01) and over time (*p* < 0.01), although no differences were detected within sampling dates (Figure 4c). The treatment × sampling date interaction was significant (*p* < 0.01).

MCH concentrations varied over time (*p* < 0.01) but did not differ between treatments overall or within sampling dates (Figure 4d). The treatment × sampling date interaction was not significant (*p* > 0.05). MCHC decreased over time (*p* < 0.01) but did not differ between treatments overall or within sampling dates (Figure 4e). The treatment × sampling date interaction was not significant (*p* > 0.05).

### 3.4. Effect of BP on Hepatic and Metabolite Biomarkers

TP, ALT, AST, and ALP concentrations were similar between treatments (*p* > 0.05; Table 1), although TP differed (*p* < 0.05) and ALT tended to vary over time (*p* = 0.07). GGT concentrations differed between treatments (*p* < 0.05). Body weight did not affect hepatic biomarkers (*p* > 0.05).

Albumin concentrations tended to differ between treatments (*p* = 0.08) and varied over time (*p* < 0.05; Table 2). Bilirubin concentrations did not differ between treatments overall (*p* > 0.05) but varied over time (*p* < 0.001) and were higher in T-CTL goats on the last two sampling dates (*p* < 0.01). The treatment × time interaction was significant (*p* < 0.01).

### 3.5. Correlations Among Blood Parameters and Parasite Burden

Supplementary Table S2 shows the correlations among blood parameters and parasitological indicators. Body weight was positively associated with MCV (*r* = 0.28) and MCH (*r* = 0.22) but negatively with MCHC (*r* = −0.19). WBC was strongly correlated with LYM (r = 0.75) and NEU (r = 0.91) and weakly correlated with MON (*r* = 0.48), confirming consistent immune activation. WBC was positively correlated to HGB (*r* = 0.17) and MCHC (*r* = 0.28). RBC and HGB (*r* = 0.87), with additional associations with MCV (*r* = 0.21), MCH (*r* = 0.53), and MCHC (*r* = 0.38). FEC showed positive association with RBC (*r* = 0.16) and tended to be associated with HGB (*r* = 0.13). FAMACHA scores were positively associated with MCV (*r* = 0.15).

### 3.6. Metabolomic Profile

We identified metabolite chemical structures using online databases (www.hmdb.ca) based on accurate masses and MS/MS fragment data. At baseline (Day-8), before treatment, we observed no significant differences between T-CTL and T-BP. PCA showed no separation between the groups across the first three principal components (*p*-values: 0.156, 0.129, 0.759), indicating similar metabolomic profiles at the outset of the study. These results suggest that before treatment, animals in both groups exhibited comparable metabolic profiles.

**Figure 5:**
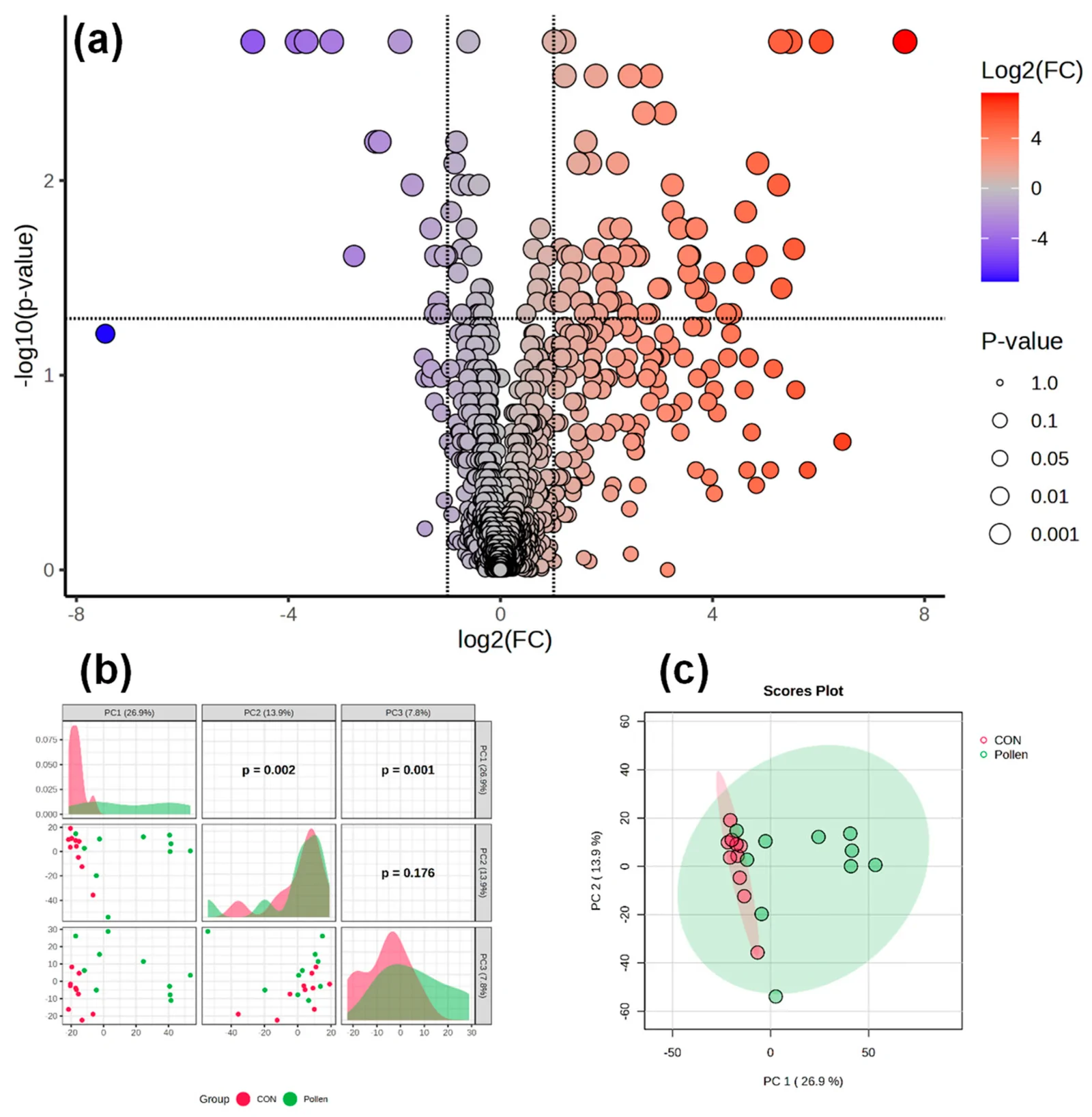
Metabolomic Profiles from goats supplemented with Bee Pollen or not after 21 days. (**a**) Volcano plot comparing T-BP (Green) and T-CTL (Red), with log2(fold change) on the x-axis and -log10(*p*-value) on the y-axis. Data points are color-coded by fold change magnitude, with red indicating increased fold changes and blue indicating reduced fold changes. Point size reflects statistical significance (16 downregulated, 70 upregulated, and 983 non-significant), with larger points corresponding to *p*-values < 0.001. A False Discovery Rate (FDR) threshold of 0.05 and a fold change threshold of 2 were applied, highlighting metabolites with significant differences. (**b**) Principal Component Analysis (PCA) of data collected on Day 21 after treatment shows separation between the T-CON and T-BP groups across the first 2 principal components (*p*-values: 0.002, 0.001, and 0.176). (**c**) PCA scores plot shows some distinction between the groups along PC1 and PC2 (PERMANOVA F-value: 8.0954, R-squared: 0.31022, *p*-value based on 999 permutations: 0.001). These findings indicate that the groups exhibited substantial differentiation 21 days after treatment.

On Day 21 (Figure 5), PCA showed separation between T-CTL and T-BP, and PERMANOVA confirmed a between-group difference (F = 8.0954, R^2^ = 0.31022, *p* = 0.001). The volcano plot identified 16 downregulated and 70 upregulated metabolites using the prespecified fold-change and FDR criteria. The differing metabolites were annotated to lipid metabolism, immune responses, and secondary metabolic pathways. Supplementary Table S3 lists the significant metabolites, along with their fold changes and *p*-values.

**Figure 6:**
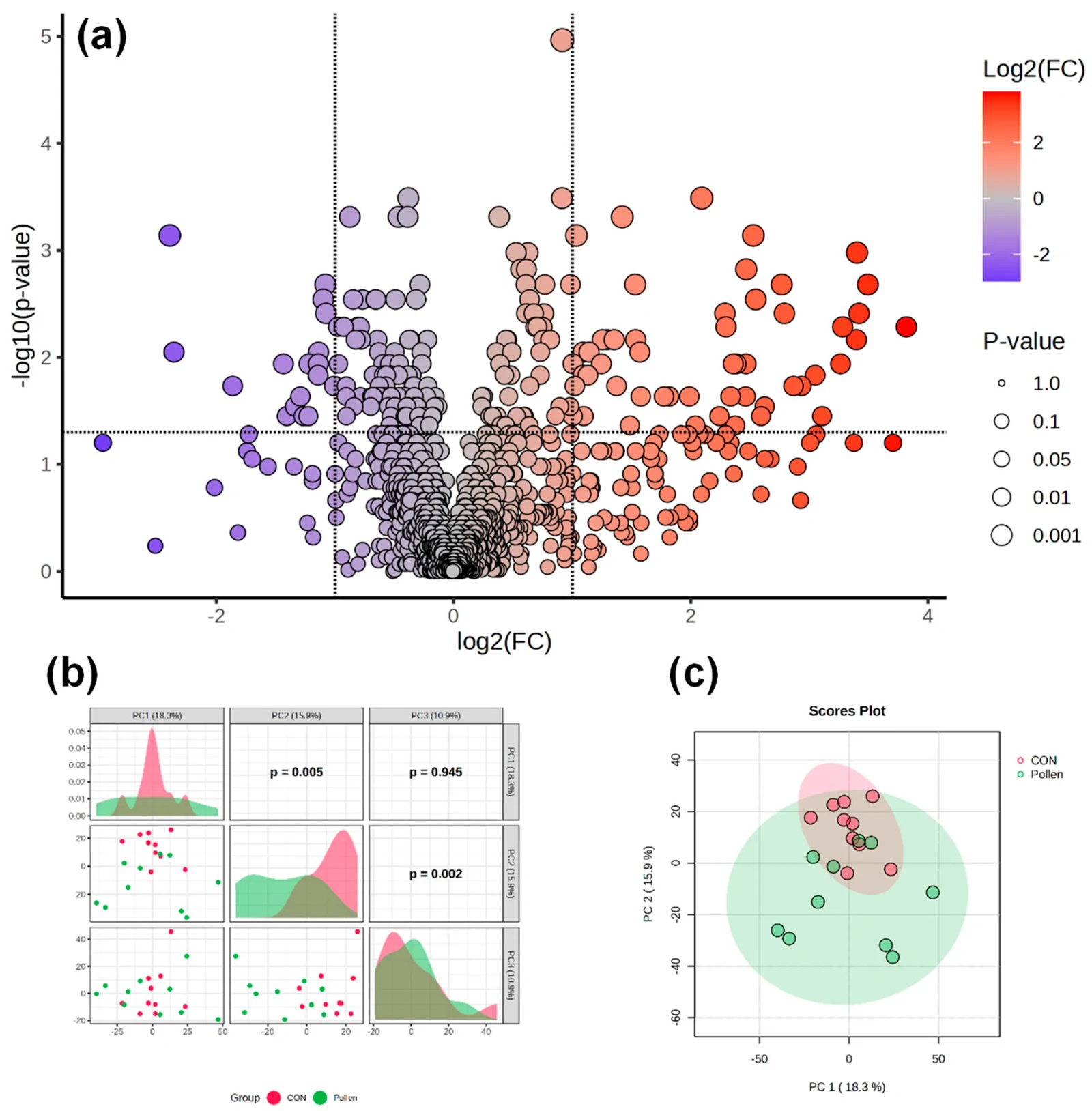
Metabolomic Profiles from goats supplemented with Bee Pollen or not after 42 days. (**a**) Volcano plot comparing Pollen and Control, with log2(fold change) on the x-axis and −log10(*p*-value) on the y-axis. Data points are color-coded by fold change magnitude, with red indicating increased fold changes and blue indicating decreased fold changes. Point size reflects statistical significance (17 downregulated, 52 upregulated, and 1000 non-significant), with larger points corresponding to *p*-values < 0.001. A False Discovery Rate (FDR) threshold of 0.05 and a fold change threshold of 2 were applied, highlighting metabolites with significant differences. (**b**) Principal Component Analysis (PCA) of data collected on Day 42 after treatment shows no clear separation between the CON and Pollen groups across the first three principal components (*p*-values: 0.002, 0.001, and 0.176). (**c**) PCA scores plot shows some distinction between the groups along PC1 and PC2 (PERMANOVA F-value: 5.4135; R-squared: 0.23121, *p*-value based on 999 permutations: 0.002). These findings indicate that the groups differed substantially 42 days after treatment.

By Day 42 (Figure 6), PCA again showed separation between T-CTL and T-BP, with PERMANOVA confirming a between-group difference (F = 5.4135, R2 = 0.23121, *p* = 0.002). The volcano plot identified 17 downregulated and 52 upregulated metabolites using the prespecified fold-change and FDR criteria. These metabolites included lipids and signaling molecules annotated to pathways related to oxidative stress, inflammatory signaling, and energy metabolism. These results indicate treatment-associated metabolomic differences rather than functional activation of the annotated pathways. Supplementary Table S4 lists the significant metabolites, and Supplementary Table S5 presents the pathway-enrichment analysis.

By Day 56, 14 days after BP supplementation had ended, no substantial metabolic differences were observed between T-CTL and T-BP. PCA showed no significant separation (*p*-values: 0.978, 0.808, 0.574), and PERMANOVA (F = 0.15334, R2 = 0.00845, *p* = 0.973) indicated no group differentiation. Taken together with the absence of baseline differences and the significant PERMANOVA results at Days 21 and 42, the time-point-specific between-group comparisons were consistent with a treatment-associated temporal pattern that was no longer detectable after supplementation ceased.

## 4. Discussion

The present study investigated the effects of bee pollen (BP) supplementation on body weight, hepatic function, systemic metabolic biomarkers, hematological status, and parasitological indicators in female goats managed under semi-arid conditions in south-central Texas. The females readily accepted and consumed the BP. Despite extensive evidence supporting the bioactive potential of BP in monogastric models, at 10 g/day, BP did not produce a consistent pattern of treatment effects across growth, hepatic, hematological, FAMACHA, or FEC outcomes. Some transient differences were observed in some CBC marker concentrations (LYM, RBC, and MCHC), suggesting that BP may exert subtle, time-dependent influences on immune and hematopoietic functions. Further, the metabolomic profile involved, specifically phospholipids, prostaglandins, and oxylipins exhibited the most notable changes throughout the study, with particularly strong differences on Days 21 and 42. These pathways are critical in mediating immune responses, inflammation, and cellular energy regulation. The metabolomic data showed no group separation at Day 56. This sequence is consistent with a treatment-associated temporal pattern during supplementation, but it is not a formal longitudinal treatment-by-time effect. Because the study evaluated only one BP dose and the metabolomic subset was small, it cannot establish dose dependence, an optimal dose, or mechanistic pathway activation. Future research should directly compare BP with other commonly used phytogenic or functional feed additives under standardized experimental conditions, including economic and practical assessments alongside physiological, immunological, and productive outcomes. This approach would clarify BP’s relative advantages and limitations and help identify the most effective and practical nutritional strategies for improving animal health and productivity.

### 4.1. Growth Performance

Body weight gain increased over time, but treatment groups did not differ. Although the T-BP group showed a numerically greater average daily gain (44 vs. 30 g/day), this difference was not statistically significant. Similarly, Fodail et al. [14] observed in Rahmani ewes that 4.5 or 9 g/day/head of BP did not increase body weight. The limited increase in body weight toward the end of the experiment, regardless of the maintenance diet, was attributed to the female goats’ limited physical activity in a pen-based experiment, as previously reported [31,32]. In contrast, results from monogastric species (rabbits) indicate that BP supplementation is associated with improved growth performance through enhanced nutrient digestibility, immune modulation, and antioxidant effects [33]. The BP dose used in this pilot trial may have been insufficient to elicit a response in body weight in goats, particularly given their larger body mass and ruminal fermentation capacity compared to smaller species such as rabbits [33]. These considerations underscore the need for species-specific optimization of BP supplementation strategies and further investigation into the role of rumen microbiota in mediating its bioactivity.

### 4.2. Hepatic Biomarkers and Liver Integrity

BP supplementation at 10 g/day did not significantly affect circulating concentrations of liver enzymes (ALT, AST, ALP, GGT), nor did it alter total protein, albumin, or bilirubin concentrations during the hepatic biomarker data do not support either hepatoprotective or hepatostimulatory under the conditions of this study.

GGT differed overall between treatments but did not vary over time, and measured values remained within the physiological range reported for healthy goats [22,34]. Mild temporal variation in other hepatic biomarkers was not accompanied by a consistent treatment pattern. Fodail et al. [14] likewise reported no effect of a similar BP dose on AST or ALT in ewes. Reports from monogastric models have described reductions in liver enzymes after BP exposure [6,35], but those findings cannot be directly extrapolated to adult ruminants because of species differences, ruminal transformation of dietary compounds, and differences in baseline health status.

Similarly, BP supplementation did not affect indicators of protein metabolism in goats. Total protein and albumin concentrations were consistent across treatment groups and sampling dates, and both remained within normal physiological limits, as defined by reference ranges typically observed in healthy goats [22,34]. In contrast, Fodail et al. [14] reported, with a similar dose to that in the current experiment, that total protein levels increased in lactating Rahmani ewes after BP supplementation, but albumin levels did not change. These findings also differ from reports in monogastrics, where BP and related bee products have been associated with enhanced protein synthesis and improved metabolic profiles [6]. Although Fodail et al. [14] observed a positive effect of BP on total proteins, this discrepancy may again reflect differences in digestive and absorptive mechanisms, as microbial degradation in the rumen may limit the systemic availability of BP-derived compounds.

Bilirubin concentrations were generally within the physiological range. A between-group difference was observed at the final sampling point, but because this was modest and limited to this time point, it should not be interpreted as evidence of hepatoprotection. Reports of antioxidant or hepatoprotective effects of BP in monogastric species [7,36].

The absence of a consistent hepatic response at 10 g/day does not establish that the dose was insufficient, because only one dose was tested. Previous ruminant studies have used different doses and supplementation durations [14,37]. Future dose-ranging studies are therefore needed to determine whether dose, duration, or delivery method modifies BP bioavailability or hepatic responses in adult goats.

### 4.3. White Blood Cells

CBC parameters remained within reported reference ranges, and the overall hematological pattern did not provide evidence of a consistent immunomodulatory effect of BP [32,38]. WBC and NEU declined over time in both groups, and their strong positive correlation is expected because neutrophils contribute to total WBC counts; this correlation should not be interpreted as evidence of immune activation. LYM differed between groups at several time points, including baseline or pre-treatment measurements (D-8 and D0), which limits treatment attribution. Elevated lymphocyte levels have been reported in goats experiencing high energy demands, such as during lactation or parasite challenge [38,39]. Consequently, we interpret the observed leukocyte differences descriptively rather than as evidence that BP stimulated adaptive immunity. In monogastric animals, stable hematological profiles have been observed when BP was supplemented with propolis [40], supporting the view that BP bioactive compounds may contribute to immunomodulation and antioxidant defense. Nevertheless, findings from monogastric species [40] remain useful for hypothesis generation but cannot establish an immunomodulatory benefit in adult goats.

### 4.4. Hematological Indices and Anemia Risk

Erythrocyte indices remained within reported reference ranges throughout the experiment [32,38]. Although transient differences in RBC and MCHC were observed at selected time points, most erythrocytic indices, including HGB, MCV, and MCH, did not show a consistent treatment pattern. Therefore, these isolated differences do not provide sufficient evidence of enhanced erythropoiesis, improved hemoglobin synthesis, or protection against anemia. Reports from broilers and rodent models [7,41,42] provide comparative context but should not be extrapolated as evidence of a hematopoietic benefit in adult goats.

### 4.5. Parasitological Parameters

FEC increased over time in both groups, consistent with changing gastrointestinal nematode egg shedding under the prevailing environmental conditions [28]. Strongyle-type eggs were the predominant category detected [29], but the McMaster method used here does not permit reliable species-level identification; therefore, these eggs cannot be attributed specifically to *H. contortus*. Nevertheless, compounds found in BP may inhibit parasite egg hatching, disrupt larval development, or enhance host immune responses, thereby reducing parasite burden [43,44]. FAMACHA scores were transiently higher in T-BP at Days 21 and 28, which indicates greater, not reduced, anemia severity at those time points [45]. FEC was lower in T-BP at one late sampling point, but the treatment × time interaction was not significant, and we observed no consistent reduction across sampling dates. Our findings highlight the complexity of host–parasite interactions and emphasize the need for species-specific evaluations when testing alternative supplements. Although the bioactive components of BP have demonstrated antiparasitic activity in other models, their efficacy in small ruminants remains poorly defined. Therefore, the utility of BP as a sustainable nutraceutical anthelmintic remains inconclusive and requires further investigation with higher doses and longer treatment durations. Such studies would clarify BP’s role in parasite management and its broader contribution to small ruminant health.

### 4.6. Correlations

The correlation analysis describes co-variation among measured traits and should not be interpreted causally. The positive associations of live weight with MCV and MCH, and its negative association with MCHC, suggest that heavier animals tend to produce larger erythrocytes with greater hemoglobin content but lower hemoglobin concentration. This pattern likely reflects improved nutritional status and increased erythropoietic activity in animals with greater body reserves, as previously reported [46,47,48]. The strong WBC-NEU correlation is expected because neutrophils contribute to total WBC counts and does not, by itself, demonstrate immune activation. Associations among RBC, HGB, MCV, MCH, and MCHC are consistent with their physiological and mathematical interdependence [47,48]. Associations between FEC and erythrocyte variables were weak and therefore do not provide sufficient evidence of compensatory erythropoiesis. Comparable findings have been reported in Pelibuey sheep, where high parasite burdens were linked to lower RBC and HGB levels [49]. Likewise, FAMACHA showed only weak or nonsignificant correlations with hematological variables. These results support cautious use of the correlation analysis as descriptive context rather than mechanistic evidence.

### 4.7. Metabolomic Signature

At Day 42, untargeted plasma metabolomics identified 52 upregulated and 17 downregulated metabolites in T-BP relative to T-CTL. These metabolites were annotated to lipid-related, endocannabinoid, retinoid, steroid, fatty acid, and arachidonic acid metabolism which are associated with lipid utilization, energy metabolism, and cellular energy homeostasis. Nevertheless, our results provide compelling indirect and correlative evidence, but further research is needed to elucidate the mechanism.

Several fatty-acid-related metabolites differed between groups, including docosapentaenoic acid (22:5n-3), palmitoleic acid, myristoleic acid, azelaic acid, sebacic acid, 8-dodecenoylcarnitine, and 5-methyloctanoyl-CoA. Medium- and long-chain acylcarnitines and dicarboxylic acids is consistent with enhanced β-oxidation, mitochondrial metabolic resilience in this study [50,51,52,53].

Differences were also observed in glucuronidated steroids, cholic acid glucuronide, glycocholic acid, taurodeoxycholic acid, and nordeoxycholic acid. Because *H. contortus* infection is characterized by hemorrhage and hypoproteinemia [27], increased bile acid turnover, improved hepatic function, enhanced erythropoiesis, or accelerated recovery from anemia [54,55,56].

Eicosanoids and other lipid mediators, including 18-HEPE, 12,13-DiHOME, 9-OxoODE, 10-nitrolinoleic acid, prostaglandins, and leukotrienes, also differed between groups. Elevated omega-3–derived mediators such as 18-HEPE and PGE_3_ are linked to pro-resolving responses, while regulated leukotriene production supports effective but controlled inflammation [57,58]. Because excessive inflammation during *H. contortus* infection damages the abomasal mucosa [27], it would be feasible to hypothesize that BP-mediated modulation of eicosanoid balance may enhance parasite-specific Th2 immunity, limit collateral tissue injury, preserve mucosal integrity, and ultimately reduce worm establishment and fecundity.

Retinoid-, vitamin D-, and sphingolipid-related metabolites also differed between groups. These metabolite classes have established roles in mucosal immune regulation. Retinoic acid promotes gut-associated lymphoid tissue development, IgA production, and epithelial differentiation [59,60], while vitamin D modulates innate immunity and macrophage and T-cell responses [61,62]. Sphingolipids are central to membrane architecture and immune cell signaling [63,64]. Therefore, it is feasible to hypothesize that these changes are likely to enhance leukocyte responsiveness, reinforce epithelial barrier defences, and promote effective parasite clearance.

Differences were also observed in metabolites, including indole-3-propionic acid, 4-ethylphenylsulfate, cynaroside A, shoyuflavone A, and hydroxymatairesinol glucuronide. Indole derivatives are associated with microbial homeostasis, mucosal biology, and redox balance [65,66]. Enhanced antioxidant capacity and microbial equilibrium may create a less permissive environment for larval development and improve host nutrient extraction efficiency.

In summary, BP supplementation reflects compelling indirect and correlative evidence integrating enhanced hepatic function and reduced gastrointestinal nematode infection in small ruminants. Nevertheless, these correlations need further mechanistic support.

## 5. Conclusions

At the tested dose and during the summer months, BP supplementation did not produce consistent treatment effects on body weight, hepatic biomarkers, hematological indices, FAMACHA scores, or FEC across the study period in crossbred Spanish × Boer non-pregnant goats maintained under subtropical conditions. Additionally, environmental conditions were atypical, and strongyle-type egg counts, presumably *Haemonchus contortus*, increased. Metabolomic profiling identified treatment-associated differences during supplementation (Days 21 and 42), but no group differences at Day 56. The differing metabolites mapped to pathways related to lipid signaling, inflammatory processes, bile-acid and retinoid metabolism, and cellular energy metabolism; however, pathway enrichment does not demonstrate functional activation or improved physiological function. Because we tested a single BP dose, we cannot establish dose–response relationships or definitive longitudinal effects. These findings should therefore be considered exploratory and hypothesis-generating and support larger dose-ranging studies with prespecified longitudinal analyses, species-resolving parasitological methods, and rigorous multivariate model validation.

## Figures and Tables

**Table 1:** Circulating concentrations of total protein (TP; ug/mL), alanine aminotransferase (ALT; ng/mL), aspartate aminotransferase (AST; ng/mL), alkaline phosphatase (ALP; ng/mL), and gamma-glutamyl transferase (GGT; ng/mL) were assessed at specific time points (days-8 [D -8], 21 [D -21], and 42 [D -42]) from female goats that received (T-BP) or not (T-CTL) BP for 42 days. Values are presented as means ± standard error of the mean (SEM).

Variable	T-CTL			T-BP			SEM	TRT	Date	TRT*Date
	D -8	D -21	D -42	D -8	D -21	D -42				
TP	3.6	1.9	2.1	3.2	1.9	2.1	0.36	0.76	0.01	0.29
ALT	0.10	0.10	0.11	0.10	0.10	0.12	0.002	0.36	0.07	0.89
AST	0.34	0.44	0.44	0.35	0.41	0.44	0.02	0.91	0.12	0.53
ALP	0.25	0.35	0.26	0.60	0.36	0.65	0.06	0.61	0.42	0.75
GGT	0.16	0.19	0.19	0.19	0.18	0.17	0.01	0.03	0.64	0.75

**Table 2:** Circulating concentrations of albumin (mg/mL) were assessed at specific time points (days-8 [D -8], 7 [D -7], 21 [D -21], and 42 [D -42]) from female goats that received (T-BP) or not (T-CTL) BP for 42 days. Values are presented as means ± standard error of the mean (SEM).

Variable	T-CTL				T-BP				SEM	TRT	Date	TRT*Date
	D -8	D -7	D -21	D -42	D -8	D -7	D -21	D -42				
Albumin	0.25	0.21	0.22	0.20	0.21	0.22	0.21	0.20	0.005	0.08	0.03	0.39

## Data Availability

The data presented in the manuscript were part of Adriana Rojas’s Thesis Project. The data that support the findings of this study are available from the corresponding author upon reasonable request.

## Supplementary Materials

The following supporting information can be downloaded at: https://www.mdpi.com/article/10.3390/biology15191717/s1, Table S1: Chemical Composition and Analytical Analysis of Bee Pollen Used in the Experiment. The goats received a pilot dose of 10 g/day/head for 49 days; Table S2: Correlations (r) among white blood cells (WBC), lymphocytes (LYM), monocytes (MON), neutrophils (NEU), red blood cells (RBC), hemoglobin (HGB), mean corpuscular volume (MCV), mean corpuscular hemoglobin (MCH), and mean corpuscular hemoglobin concentration (MCHC), fecal counts (FECs), and FAMACHA scores (FAM). The data combined groups; Table S3: List of significant metabolites identified by the volcano plot of T-BP vs. T-CTL on Day 21. T-BP goats received Bee Pollen supplement for 21 days; Table S4: List of significant metabolites identified by the volcano plot of T-BP vs. T-CTL on Day 42. T-BP goats received Bee Pollen supplement for 42 days; Table S5: Lists the enrichment analysis pathway for perturbed metabolites expressed on Days 21 and 42. X indicates if the pathway was expressed on Day 21 or 42.
