# Supplementary material for: Metabolomic Profiling Uncovers Physiological and Parasitological Adaptations to Bee Pollen Supplementation in Goats
Source: Biology (Basel). 2026 Sep 29;15(19):1717. doi: 10.3390/biology15191717 (PMC13663435; doi:10.3390/biology15191717)
Supplement: Supplementary file 1 [file biology-15-01717-s001.zip › biology-4561830-supplementary.pdf]

Table S1. Chemical Composition and Analytical Analysis of Bee Pollen Used in the Experiment. The goats received a pilot dose of 10 g/day/head for 49 days.

Chemical Analysis		
**Item (unit)**	**Dry Basis**	**As Received**
Moisture (%)		6.86
Dry Matter (%)		93.14
Crude Protein (%)	22.45	20.91
Crude Fiber (%)	4.72	4.40
ADF-Acid Detergent Fiber (%)	0.90	0.84
NEL: Net Energy-Lactation (Mcal/lb)	1.03	0.96
NEG: Net Energy-Gain (Mcal/lb)	0.83	0.77
NEM: Net Energy-Maintenance (Mcal/lb)	1.16	1.08
TDN: Total Digestible Nutrients (%)	97.80	91.09
Fat (EE; %)	6.36	5.92
Ash (%)	2.62	2.44
NFE-Nitrogen Free Extract (%)	63.85	
Carbohydrates Per 100 Grams (grams)	69	64
Calcium (%)	0.11	0.10
Phosphorus (%)	0.47	0.44
Potassium (%)	0.49	0.46
Magnesium (%)	0.09	0.08
Sodium (%)	0.03	0.03
Sulfur (%)	0.27	0.25
Aluminum (ppm)	103.00	95.93
Cobalt (ppm)	Less than 0.2	

Copper (ppm)	9.37	8.73
Iron (ppm)	122.00	113.63
Manganese (ppm)	20.50	19.09
Molybdenum (ppm)	Less than 0.3	
Zinc (ppm)	40.70	37.91
pH (s.u.)		4.49
**Analytical Analysis**		
Lactic Acid (%)		0.14
Acetic Acid (%)		1.49
Butyric Acid (%)		Less than 0.01
Propionic Acid (%)		Less than 0.01
Valeric Acid (ppm)		0.01
Vitamin A (IU/lb)		Less than 15
Vitamin D3 (IU/lb)		4610
Vitamin E (IU/lb)		49
C 04:0 - Methyl Butyrate (%)		Less than 0.1
C 05:0 - Methyl Pentanoate (%)		Less than 0.1
C 06:0 - Methyl Caproate (%)		Less than 0.1
C 07:0 - Methyl Heptanoate (%)		Less than 0.1
C 08:0 - Methyl Caprylate (%)		1.1
C 09:0 - Methyl Nonanoate (%)		Less than 0.1
C 10:0 - Methyl Caprate (%)		Less than 0.1
C 11:0 - Methyl Undecanoate (%)		Less than 0.1
C 12:0 - Methyl Laurate (%)		1.5
C 12:1 - Methyl Dodecenoate (%)		Less than 0.1

C 13:0 - Methyl Tridecamoate (%)	Less than 0.1
C 13:1 - Methyl Tridecenoate (%)	Less than 0.1
C 14:0 - Methyl Myristate (%)	1.6
C 14:1 - Methyl Myristoleate (%)	Less than 0.1
C 15:0 - Methyl Pentadecanoate (%)	Less than 0.1
C 15:1 - Methyl Pentadecenoate (%)	Less than 0.1
C 16:0 - Methyl Palmitate (%)	29.0
C 16:1 - Methyl Palmitoleate (%)	Less than 0.1
C 17:0 - Methyl Heptadecanoate (%)	0.8
C 17:1 - Methyl Heptadecanoleate (%)	Less than 0.1
C 18:0 - Methyl Stearate (%)	1.7
C 18:1 - Methyl Oleate (%)	7.8
C 18:2 - Methyl Linoleate (%)	14.9
C 18:3 - Methyl Linolenate (%)	25.2
C 20:0 - Methyl Arachidate (%)	Less than 0.1
C 20:1 - Methyl Eicosenoate (%)	2.3
C 20:2 - Methyl Eicosadienoate (%)	Less than 0.1
C 20:3 - Methyl Eicosatrienoate (%)	Less than 0.1
C 20:4 - Methyl Arachidonate (%)	Less than 0.1
C 20:5 - Methyl Eicosapentaenoate (EPA; %)	2.2
C 22:0 - Methyl Behenate (%)	Less than 0.1
C 22:1 - Methyl Erucate (%)	Less than 0.1
C 22:2 - Methyl Docosadienoate (%)	Less than 0.1
C 22:6 - Methyl Docosahexaenoate (%)	Less than 0.1
C 24:0 - Methyl Lignocerate (%)	Less than 0.1

C 24:1 - Methyl Nervonate (%)	Less than 0.1
**Amino Acids Profile**	
Aspartic acid (%)	2.42
Threonine (%)	0.79
Serine (%)	1.14
Glutamic acid (%)	2.37
Proline (%)	1.63
Glycine (%)	1.15
Alanine (%)	1.26
Cystine (%)	0.45
Valine (%)	0.76
Methionine (%)	0.39
Isoleucine (%)	0.79
Leucine (%)	1.38
Tyrosine (%)	0.66
Phenylalanine (%)	0.73
Lysine (%)	1.04
Histidine (%)	0.42
Arginine (%)	0.66
Tryptophan (%)	0.25

Table S2. Correlations (*r*) among white blood cells (WBC), lymphocytes (LYM), monocytes (MON), neutrophils (NEU), red blood cells (RBC), hemoglobin (HGB), mean corpuscular volume (MCV), mean corpuscular hemoglobin (MCH), and mean corpuscular hemoglobin concentration (MCHC), fecal counts (FECs), and FAMACHA scores (FAM). The data combined groups.

	LWT	WBC	LYM	MON	NEU	RBC	HGB	MCV	MCH	MCHC	FECs	FAM
LWT	NA	NS (-0.1)	NS (0.01)	NS (0.06)	0.06 (-0.14)	0.03 (-0.17)	NS (-0.02)	0.001 (0.28)	0.005 (0.22)	0.01 (-0.19)	NS (-0.01)	0.06 (-0.14)
WBC		NA	< 0.001 (0.76)	< 0.001 (0.48)	< 0.001 (0.91)	0.07 (0.14)	0.03 (0.17)	NS (-0.04)	NS (0.11)	< 0.001 (0.28)	NS (-0.1)	NS (0.09)
LYM			NA	< 0.001 (0.50)	< 0.001 (0.42)	0.01 (0.21)	< 0.001 (0.32)	0.05 (0.15)	< 0.001 (0.28)	0.04 (0.16)	NS (-0.02)	NS (0.04)
MON				NA	< 0.001 (0.33)	NS (0.06)	NS (0.08)	NS (0.03)	NS (0.06)	NS (0.03)	NS (-0.08)	NS (0.04)
NEU					NA	NS (0.06)	NS (0.04)	0.06 (-0.15)	NS (-0.01)	0.001 (0.29)	NS (-0.13)	NS (0.09)
RBC						NA	< 0.001 (0.87)	0.004 (-0.22)	NS (0.04)	< 0.001 (0.52)	0.03 (0.16)	NS (-0.01)
HGB							NA	0.005 (0.22)	< 0.001 (0.53)	< 0.001 (0.38)	0.09 (0.13)	NS (0.13)
MCV								NA	< 0.001 (0.83)	< 0.001 (-0.59)	NS (-0.06)	0.05 (0.15)
MCH									NA	NS (-0.11)	NS (-0.04)	NS (0.13)
MCHC										NA	NS (0.01)	NS (-0.11)
FECs											NA	NS (-0.05)
FAM												NA

Table S3. List of significant metabolites identified by the volcano plot of T-BP vs. T-CTL on Day 21. T-BP goats received Bee Pollen supplement for 21 days.

	**FC**	**log2(FC)**	**adjusted P-value**	**-log10(p)**
DG(8:0/20:5-3OH(5,6,15))	198.04	7.6297	0.0019287	2.7147
p-HPEA-EDA	66.354	6.0521	0.0019287	2.7147
Cyclo(glycylleucylvalylleucylprolylseryl)	44.184	5.4654	0.0019287	2.7147
Hymenoxon	38.909	5.282	0.0019287	2.7147
Nuzhenide	0.039237	-4.6716	0.0019287	2.7147
DG(PGJ2/2:0)	0.070075	-3.835	0.0019287	2.7147
Austalide G	0.078799	-3.6657	0.0019287	2.7147
1,8,11-Heptadecatriene	0.11001	-3.1843	0.0019287	2.7147
1,4-Dihydroxy-2-naphthoic acid	0.26866	-1.8962	0.0019287	2.7147
20-Trihydroxy-leukotriene-B4	2.2902	1.1955	0.0019287	2.7147
12,13-DiHOME	2.0194	1.014	0.0019287	2.7147
Dodecadienoic acid	7.1312	2.8341	0.002893	2.5387
18-Hydroxyeicosapentaenoic acid	5.4366	2.4427	0.002893	2.5387
N-Undecanoylglycine.1	3.4803	1.7992	0.002893	2.5387
PE-NMe2(16:0/20:1)	2.308	1.2067	0.002893	2.5387
Psilostachyin	8.5566	3.097	0.0045002	2.3468
Cholestane-3,7,12,25-tetrol-3-glucuronide	6.529	2.7069	0.0045002	2.3468
Dibutyl sulfosuccinate	0.19599	-2.3512	0.006312	2.1998
Mupirocin	0.20617	-2.2781	0.006312	2.1998
3-Oxo-alpha-ionol	3.0463	1.607	0.006312	2.1998
N-Stearoyl Asparagine	28.847	4.8504	0.0081433	2.0892
Ponasteroside A	4.6251	2.2095	0.0081433	2.0892
N-Undecanoylglycine	3.2387	1.6954	0.0081433	2.0892
Myristoleic acid	2.7615	1.4655	0.0081433	2.0892
Docosapentaenoic acid (22n-3)	37.906	5.2443	0.01052	1.978
4-Ketoretinoic acid glucuronide	9.4675	3.243	0.01052	1.978
Etiocholanolone glucuronide	0.31543	-1.6646	0.01052	1.978
Sebacic acid	24.655	4.6238	0.014465	1.8397

MG(5-iso PGF2VI)	9.5624	3.2574	0.014465	1.8397
Cynaratriol	12.963	3.6963	0.017621	1.754
10-Nitrolinoleic acid.1	12.69	3.6657	0.017621	1.754
O-Desmethylvenlafaxine glucuronide	10.381	3.3758	0.017621	1.754
2-Aminooctadeca-4,14-diene-1,3-diol	4.8218	2.2696	0.017621	1.754
Rhubafuran	4.1409	2.05	0.017621	1.754
Caffeine	0.40119	-1.3176	0.017621	1.754
Cervonoyl ethanolamide	46.394	5.5359	0.02245	1.6488
9,10-Dihydroxystearic acid	5.422	2.4388	0.02245	1.6488
Trilostane	3.4026	1.7666	0.02245	1.6488
O-Arachidonoyl Ethanolamine	2.2125	1.1457	0.02245	1.6488
1,25-Dihydroxy-16-ene-vitamin D3	28.475	4.8316	0.024371	1.6131
Hexadecasphingosine	11.929	3.5763	0.024371	1.6131
12-oxo-PDA	11.675	3.5454	0.024371	1.6131
Glabric acid	0.14737	-2.7625	0.024371	1.6131
10-Nitrolinoleic acid	6.1	2.6088	0.024371	1.6131
9-OxoODE	5.831	2.5437	0.024371	1.6131
DG(8:0/TXB2)	5.1264	2.3579	0.024371	1.6131
3-Oxocholic acid	4.1858	2.0655	0.024371	1.6131
Dihydrocapsaicin	4.1573	2.0557	0.024371	1.6131
Sphinganine	2.5488	1.3498	0.024371	1.6131
Rimantadine	2.4046	1.2658	0.024371	1.6131
13-Retinoic acid	0.4263	-1.23	0.024371	1.6131
Creatinine	0.48403	-1.0468	0.024371	1.6131
Isoquinoline	0.49694	-1.0089	0.024371	1.6131
16-B1-phytoprostane	24.054	4.5882	0.029779	1.5261
Cholic acid glucuronide	16.411	4.0366	0.029779	1.5261
N-Decanoylglycine	11.699	3.5484	0.029779	1.5261
1,10-Bisaboladiene-3,4-diol	4.3849	2.1325	0.029779	1.5261
3-Oxo-4,6-choladienoic acid	3.8096	1.9296	0.029779	1.5261
Dehydrophytosphingosine	2.8244	1.4979	0.029779	1.5261
Homophytanic acid	2.5109	1.3282	0.029779	1.5261
Azelaic acid	39.475	5.3029	0.035793	1.4462
Kahweol	13.278	3.731	0.035793	1.4462

Ginkgoic acid	12.762	3.6738	0.035793	1.4462
Nandrolone	8.104	3.0186	0.035793	1.4462
Hexaminolevulinate	7.8789	2.978	0.035793	1.4462
19-Norandrostenedione	3.9911	1.9968	0.035793	1.4462
Lucidenic acid I	2.4498	1.2927	0.035793	1.4462
Icaridin	14.727	3.8804	0.041963	1.3771
PS(20:2/PGF1alpha)	4.8852	2.2884	0.041963	1.3771
2-Heptadecylfuran	4.2422	2.0848	0.041963	1.3771
Levonorgestrel	4.1002	2.0357	0.041963	1.3771
PI(TXB2/20:3)	2.9164	1.5442	0.041963	1.3771
PG(i-19:0/PGE2)	2.8494	1.5107	0.041963	1.3771
Indole-3-propionic acid	0.44372	-1.1723	0.041963	1.3771
Palmitoleic acid	2.193	1.1329	0.041963	1.3771
Alpha-Linolenoyl ethanolamide	2.0086	1.0062	0.041963	1.3771
2-Hydroxydecanedioic acid	20.532	4.3598	0.048301	1.316
PE(20:5-OH(18)/18:0)	19.116	4.2567	0.048301	1.316
Esmolol	7.0522	2.8181	0.048301	1.316
Dodecatrienoic acid	6.753	2.7555	0.048301	1.316
8-Amino-7-oxononanoic acid	5.2907	2.4034	0.048301	1.316
Cynaroside A.1	3.5721	1.8368	0.048301	1.316
Cynaroside A	3.3008	1.7228	0.048301	1.316
3a,6b,7b-Trihydroxy-5b-cholanoic acid	2.9998	1.5849	0.048301	1.316
Indolylacryloylglycine.1	0.42225	-1.2438	0.048301	1.316
N,N-Dimethylsphingosine	0.45528	-1.1352	0.048301	1.316

FC = fold change; log2(FC) = logarithmic fold change; raw.pval = raw p-value; -log10(p) = negative logarithm (base 10) of the p-value. A False Discovery Rate (FDR) threshold of 0.05 and a fold change threshold of 2 were applied to highlight metabolites with significant differences.

Table S4. List of significant metabolites identified by the volcano plot of T-BP vs. T-CTL on Day 42. T-BP goats received Bee Pollen supplement for 42 days.

	**FC**	**log2(FC)**	**raw.pval**	**-log10(p)**
2,5-Di-tert-butylhydroquinone	4.2649	2.0925	0.00032475	3.4884

20-Trihydroxy-leukotriene-B4	2.6781	1.4212	0.00048713	3.3124
Ponasteroside A	5.765	2.5273	0.00072528	3.1395
Glabric acid	0.19033	-2.3934	0.00072528	3.1395
Cucurbitacin I	2.05	1.0356	0.00072528	3.1395
1,25-Dihydroxyvitamin D3-26,23-lactone	10.569	3.4018	0.00105	2.9788
Kanokoside A.1	5.5308	2.4675	0.0015047	2.8226
Cholestane-3,7,12,25-tetrol-3-glucuronide	11.257	3.4928	0.0020892	2.68
Cynaroside A.1	6.8039	2.7664	0.0020892	2.68
Prostaglandin F3a	2.8907	1.5314	0.0020892	2.68
5-Methyloctanoyl-CoA	0.47251	-1.0816	0.0020892	2.68
Shoyuflavone A	5.8469	2.5477	0.0028795	2.5407
2-[6-ulfooxy)naphthalen-2-yl]propanoic acid	0.46681	-1.0991	0.0028795	2.5407
Macrocarpal I	10.695	3.4188	0.0038862	2.4105
Cynaroside A	6.9142	2.7896	0.0038862	2.4105
18-Hydroxyeicosapentaenoic acid	4.8835	2.2879	0.0038862	2.4105
4-Hydroxy-5-(phenyl)-valeric acid-O-sulphate	0.47409	-1.0768	0.0038862	2.4105
Cholic acid glucuronide	14.101	3.8177	0.005196	2.2843
Epothilone B	9.7201	3.281	0.005196	2.2843
rac-4-Hydroxy-4-O-(betaglucuronide)-all-retinyl acetate	4.9118	2.2963	0.005196	2.2843
Ganoderic acid theta	10.529	3.3963	0.0068415	2.1649
Methyl 20-dihydroprednisolonate	2.8974	1.5348	0.0068415	2.1649
Lusitanicoside	2.5561	1.354	0.0068415	2.1649
Macrophorin D	2.4534	1.2948	0.0068415	2.1649
1-Phenyl-1-cyclohexene	2.3861	1.2546	0.0068415	2.1649
PGP(22:6-OH(7)/i-22:0)	0.19504	-2.3582	0.0089307	2.0491
Glycocholic acid.1	2.9688	1.5699	0.0089307	2.0491
Leukotriene D4	2.2073	1.1423	0.0089307	2.0491
4-oxo-Retinoic acid	0.45397	-1.1393	0.0089307	2.0491
6-Epi-7-isocucurbic acid glucoside	2.1311	1.0916	0.0089307	2.0491
DG(8:0/TXB2)	9.5992	3.2629	0.011496	1.9394
2,4-Dihydroxy-6-(8-pentadecenyl)benzoic acid	5.5249	2.466	0.011496	1.9394
10,11-Dihydro--hydroxy-leukotriene E4	5.2148	2.3826	0.011496	1.9394
4-Ethylphenylsulfate	5.1279	2.3584	0.011496	1.9394
Nordeoxycholic acid	0.36981	-1.4351	0.011496	1.9394

Lubiminol	2.3721	1.2462	0.011496	1.9394
Taurodeoxycholic acid	0.42898	-1.221	0.011496	1.9394
LysoPS(18:2)	0.45347	-1.1409	0.011496	1.9394
DG(2:0/20:4-OH(20))	2.0092	1.0066	0.011496	1.9394
(1alpha,3beta,)-Pubescenin	8.277	3.0491	0.01469	1.833
Taurodeoxycholic acid.1	0.45272	-1.1433	0.01469	1.833
Prostaglandin E3.1	2.1919	1.1322	0.01469	1.833
LysoPC(20:0).1	2.1299	1.0908	0.01469	1.833
Methylprednisolone hemisuccinate	7.6371	2.933	0.018543	1.7318
Dhv-PGE2	7.2922	2.8664	0.018543	1.7318
8-Dodecenoylcarnitine	0.27495	-1.8628	0.018543	1.7318
4-Hydroxyandrostenedione glucuronide	2.5917	1.3739	0.018543	1.7318
LysoPC(20:0)	2.0307	1.022	0.018543	1.7318
tetranor-PGFM	0.4975	-1.0072	0.018543	1.7318
Adhumulinic acid	5.5123	2.4626	0.023231	1.6339
PA(22:5O(16,17)/10:0)	5.0491	2.336	0.023231	1.6339
Perindoprilat glucuronide.1	3.9653	1.9874	0.023231	1.6339
O-Desmethylvenlafaxine glucuronide	3.5236	1.817	0.023231	1.6339
MG(5-iso PGF2VI)	3.4193	1.7737	0.023231	1.6339
Tyramine glucuronide	2.9865	1.5785	0.023231	1.6339
6-Ketomyristic acid	0.40841	-1.2919	0.023231	1.6339
Thiocolchicoside	6.159	2.6227	0.028806	1.5405
Carvone	0.39558	-1.338	0.028806	1.5405
Gein	8.6149	3.1068	0.035463	1.4502
Hydroxymatairesinol glucuronide 1	6.0322	2.5927	0.035463	1.4502
Tetradecanol	4.7282	2.2413	0.035463	1.4502
MG(14:1)	0.37714	-1.4068	0.035463	1.4502
N-Eicosapentaenoyl Tyrosine	0.41606	-1.2651	0.035463	1.4502
Menthyl pyrrolidone carboxylate	0.42773	-1.2252	0.035463	1.4502
Hypochoeroside A.1	2.1402	1.0977	0.035463	1.4502
Succinylacetone	2.0072	1.0052	0.035463	1.4502
Prodolic acid	5.1859	2.3746	0.043257	1.3639
1,1'-Ethylidenebistryptophan	4.1081	2.0385	0.043257	1.3639
6-Ketoprostaglandin E1.1	2.802	1.4865	0.043257	1.3639

FC = fold change; log2(FC) = logarithmic fold change; raw.pval = raw p-value; -log10(p) = negative logarithm (base 10) of the p-value. A False Discovery Rate (FDR) threshold of 0.05 and a fold change threshold of 2 were applied to highlight metabolites with significant differences.

Table S5. Lists the enrichment analysis pathway for perturbed metabolites expressed on Days 21 and 42. X indicates if the pathway was expressed on Day 21 or 42.

Pathway	Day 21	Day 42
Adipocytokine signaling pathway	X	X
Adrenergic signaling in cardiomyocytes	X	X
African trypanosomiasis	X	X
AGE-RAGE signaling pathway in diabetic complications	X	X
Aldosterone synthesis and secretion	X	X
alpha-Linolenic acid metabolism		X
Amoebiasis	X	X
Apelin signaling pathway	X	X
Arachidonic acid metabolism	X	X
Ascorbate and aldarate metabolism	X	X
Asthma	X	
Autophagy - animal	X	
Autophagy - other	X	
B cell receptor signaling pathway	X	X
Bile secretion	X	X
Biosynthesis of amino acids	X	
Biosynthesis of cofactors	X	X
Biosynthesis of unsaturated fatty acids	X	
Biotin metabolism	X	
Caffeine metabolism	X	
Calcium signaling pathway	X	X
cAMP signaling pathway	X	X
Carbohydrate digestion and absorption	X	X
Carbon metabolism		X
Chemokine signaling pathway		
Cholesterol metabolism		X
Choline metabolism in cancer	X	X

Cholinergic synapse	X	X
Circadian entrainment	X	X
Coronavirus disease - COVID-19	X	X
C-type lectin receptor signaling pathway	X	X
Diabetic cardiomyopathy	X	X
Dopaminergic synapse	X	X
Efferocytosis	X	X
EGFR tyrosine kinase inhibitor resistance	X	X
Endocrine and other factor-regulated calcium reabsorption	X	X
ErbB signaling pathway	X	X
Estrogen signaling pathway	X	X
Fat digestion and absorption	X	X
Fatty acid biosynthesis	X	X
Fc epsilon RI signaling pathway	X	X
Fc gamma R-mediated phagocytosis	X	X
Gap junction	X	X
Gastric acid secretion	X	X
Glioma	X	X
Glutamatergic synapse	X	X
Glycerolipid metabolism	X	X
Glycerophospholipid metabolism	X	X
Glycine, serine and threonine metabolism		X
Glycosylphosphatidylinositol (GPI)-anchor biosynthesis	X	
Glyoxylate and dicarboxylate metabolism		X
GnRH secretion	X	X
GnRH signaling pathway	X	X
Growth hormone synthesis, secretion and action	X	X
Hepatocellular carcinoma	X	X
HIF-1 signaling pathway	X	X
Human cytomegalovirus infection	X	X
Human immunodeficiency virus 1 infection	X	X
Human papillomavirus infection	X	X
Inflammatory mediator regulation of TRP channels	X	X
Inositol phosphate metabolism	X	X
Insulin resistance	X	X
Insulin secretion	X	X
Kaposi sarcoma-associated herpesvirus infection	X	X

Leishmaniasis	X	X
Linoleic acid metabolism	X	X
Lipoic acid metabolism		X
Long-term depression	X	X
Long-term potentiation	X	X
MAPK signaling pathway	X	X
Melanogenesis	X	X
Natural killer cell mediated cytotoxicity	X	X
Neuroactive ligand-receptor interaction	X	X
Neurotrophin signaling pathway	X	X
Neutrophil extracellular trap formation	X	X
NF-kappa B signaling pathway	X	X
Non-small cell lung cancer	X	X
Oxytocin signaling pathway	X	X
Pancreatic cancer	X	
Pancreatic secretion	X	X
Parathyroid hormone synthesis, secretion and action	X	X
Parkinson disease	X	X
PD-L1 expression and PD-1 checkpoint pathway in cancer	X	X
Pentose and glucuronate interconversions	X	X
Pentose phosphate pathway		X
Phenylalanine, tyrosine and tryptophan biosynthesis	X	
Phosphatidylinositol signaling system	X	
Platelet activation	X	X
PPAR signaling pathway	X	X
Primary bile acid biosynthesis	X	
Rap1 signaling pathway		X
Ras signaling pathway	X	X
Regulation of actin cytoskeleton	X	X
Regulation of lipolysis in adipocytes	X	
Relaxin signaling pathway	X	X
Renin secretion	X	X
Retinol metabolism	X	X
Retrograde endocannabinoid signaling		X
Rheumatoid arthritis	X	X
s in cancer	X	X
s of neurodegeneration - multiple diseases	X	X

Salivary secretion	X	X
Salmonella infection	X	X
Serotonergic synapse	X	
Sphingolipid signaling pathway	X	X
Spinocerebellar ataxia	X	X
Starch and sucrose metabolism	X	X
Steroid hormone biosynthesis		X
T cell receptor signaling pathway	X	
Th1 and Th2 cell differentiation	X	X
Th17 cell differentiation	X	X
Thermogenesis	X	X
Thyroid hormone signaling pathway	X	X
Thyroid hormone synthesis	X	X
Tuberculosis	X	X
Ubiquinone and other terpenoid-quinone biosynthesis	X	
Vascular smooth muscle contraction	X	X
VEGF signaling pathway	X	X
Vitamin digestion and absorption	X	X
